# Fatigue in Middle-Aged and Older Adults with Axial Spondyloarthritis: A Sex-Stratified Case–Control Study

**DOI:** 10.3390/jcm15114305

**Published:** 2026-06-02

**Authors:** Joan M. Nolla, Diego Benavent, Lidia Valencia-Muntalà, Manuela González-Aguila, Blanca Alonso-Palao, Carmen Gómez-Vaquero, Javier Narváez, Xavier Juanola, Laura Berbel-Arcobé

**Affiliations:** Department of Rheumatology, IDIBELL-Hospital Universitari de Bellvitge, University of Barcelona, 08907 Barcelona, Spain; diegobenavent@bellvitgehospital.cat (D.B.); lvalencia@bellvitgehospital.cat (L.V.-M.); manuela.gonzalez@bellvitgehospital.cat (M.G.-A.); blancaalonso@bellvitgehospital.cat (B.A.-P.); carmen.gomez@bellvitgehospital.cat (C.G.-V.); fjnarvaez@bellvitgehospital.cat (J.N.); xjuanola@gmail.com (X.J.); lberbel@bellvitgehospital.cat (L.B.-A.)

**Keywords:** axial spondyloarthritis, fatigue, FACIT-F, sex differences, patient-reported outcomes, case–control study, older adults

## Abstract

**Background:** Fatigue is a common and disabling symptom in axial spondyloarthritis (axSpA), yet its magnitude relative to the general population and potential sex-specific differences remain insufficiently characterized, particularly in older adults. We therefore aimed to assess fatigue in adults aged ≥ 50 years with axSpA, using the Functional Assessment of Chronic Illness Therapy–Fatigue (FACIT-F) scale, to compare fatigue levels with age- and sex-matched controls, and to explore sex-specific differences and clinical factors associated with fatigue. **Methods:** We conducted an observational case–control study including consecutive patients with axSpA aged ≥ 50 years and control subjects frequency-matched by age and sex. Fatigue was assessed using the FACIT-F, and clinically relevant fatigue was defined as a FACIT-F score < 40. Case–control comparisons were stratified by sex, and sex-stratified multivariable linear regression models were applied. **Results:** The study included 173 patients with axSpA (120 men, 53 women; mean age: 64.2 years) and 383 controls. Clinically relevant fatigue was more frequent in women than in men (84.9% vs. 50.0%; *p* < 0.001). Women reported more severe fatigue than men (FACIT-F: 29.4 ± 10.4 vs. 37.4 ± 10.2; *p* < 0.001). In case–control comparisons, fatigue was greater in patients than in controls in both sexes, with descriptively larger differences among women. In sex-stratified multivariable analyses, the ASAS Health Index (ASAS-HI) was independently associated with fatigue in both men and women. In reduced models including age, BASDAI, and ASAS-HI, ASAS-HI remained independently associated with FACIT-F in both men (β: −1.74, 95% CI: −2.08 to −1.41) and women (β: −1.80, 95% CI: −2.35 to −1.26; *p* < 0.001 for both). BASDAI showed an additional independent association in women (β: −1.19, 95%: CI −2.09 to −0.30; *p* = 0.010), but not in men. **Conclusions:** Fatigue is highly prevalent and clinically relevant in adults aged ≥50 years with axSpA, with a clear sex-specific pattern. Women experience a greater fatigue burden, and comparisons with controls suggest a larger excess among women. Fatigue represents an important dimension of disease burden in axSpA, with stronger associations with overall health status than with conventional inflammatory measures.

## 1. Introduction

Fatigue is a common and disabling symptom in several chronic inflammatory rheumatic diseases [1]. It has been described as a persistent sense of physical and mental exhaustion that interferes with daily activities and quality of life [2]. Beyond its high prevalence, fatigue is associated with impaired functional status, reduced participation, and poorer health-related quality of life in patients with inflammatory rheumatic conditions [1,2]. Importantly, fatigue is increasingly understood as a multidimensional construct encompassing physical, cognitive, and emotional components, which are only partly reflected by conventional clinical assessments [1,2]. In the general population, estimates of fatigue prevalence vary substantially, reflecting its subjective nature and methodological heterogeneity across studies [3]. Such variability poses a challenge when attempting to distinguish disease-related fatigue from background levels across middle and older adulthood.

Axial spondyloarthritis (axSpA) is a chronic inflammatory disease primarily affecting the axial skeleton and characterized by pain, stiffness, and functional limitations [4]. With increasing age, axSpA often coexists with accumulated structural damage, comorbidities, and functional decline, which may amplify symptom burden and alter the relationship between inflammatory activity and patient-reported outcomes [5]. In the present study, we focused on adults aged 50 years and older, a clinically relevant stage spanning later midlife and older adulthood. In this age range, the interpretation of fatigue is more likely to be influenced by the combined effects of axSpA, comorbidity, and age-related functional decline. In this context, non-inflammatory drivers of symptoms may play an increasingly prominent role, particularly those factors not directly reflected by inflammatory markers [5,6]. Despite major therapeutic advances and improved control of inflammation, a substantial proportion of patients continue to report residual symptoms [6]; among these, fatigue is one of the most frequently reported and clinically relevant complaints. These observations suggest that fatigue may represent a domain of disease impact that is not adequately addressed by current treatment strategies focused primarily on inflammation [6,7].

Although fatigue has been widely described in axSpA [7], important gaps remain in the available evidence. Most studies are cross-sectional and restricted to within-cohort analyses while comparative data remain scarce. Furthermore, the absence of appropriate control populations limits the interpretation of fatigue severity and hinders the estimation of disease-attributable burden. As a result, the extent to which fatigue observed in axSpA patients exceeds background levels expected in the general population is not well defined, particularly when age-related contributors may influence fatigue severity. This issue is particularly pertinent in middle-aged and older adults, in whom the use of external comparators may improve the interpretation of fatigue burden.

Sex-related differences represent an additional underexplored dimension. Increasing evidence across inflammatory rheumatic diseases suggests that women and men may differ in symptom burden and patient-reported outcomes. In axSpA [8], sex differences have been described for disease characteristics and treatment response, yet fatigue has rarely been examined from a sex-specific perspective, particularly across middle and older adults. These differences are likely to be multifactorial, although their underlying determinants remain incompletely understood. Consequently, potential differences in fatigue burden between women and men with axSpA remain poorly characterized.

The assessment of fatigue is further complicated by the use of heterogeneous measurement tools [9]. This heterogeneity limits comparability across studies and highlights the need for instruments with strong psychometric performance and clear clinical interpretability. The Functional Assessment of Chronic Illness Therapy–Fatigue (FACIT-F) scale [10,11] is a validated instrument with good psychometric performance in axSpA and is being increasingly used in clinical trials [12,13]. In addition to its robustness, the FACIT-F provides clinically interpretable thresholds that facilitate the identification of patients with meaningful fatigue burdens and enable comparisons across populations.

In this context, the present study aimed to assess fatigue in middle-aged and older adults with axSpA, using the FACIT-F scale, to compare fatigue levels with those of an age- and sex-matched control population, and to examine sex-specific differences in fatigue burden. By incorporating an external comparator and a sex-stratified analytical approach, we sought to provide a more precise estimate of the excess fatigue associated with axSpA in middle-aged and older adults. In addition, we evaluated the association between fatigue and key disease-related variables in this population.

## 2. Materials and Methods

### 2.1. Study Population

We conducted an observational, case–control study including individuals aged ≥50 years. Cases were patients that met the Assessment of Spondyloarthritis International Society (ASAS) classification criteria for axSpA [14,15], who were consecutively recruited during routine outpatient visits at the Department of Rheumatology of Bellvitge University Hospital, a tertiary academic center. Recruitment took place from February 2023 to November 2025. The study focused on adults aged 50 years and older in order to examine fatigue in a clinically relevant age range spanning later midlife and older adulthood, in which comorbidity burden, functional decline, and background fatigue may complicate the interpretation of fatigue associated with axSpA.

Controls were drawn from a hospital-based population without inflammatory arthritis and were frequency-matched to cases based on age and sex. Individual 1:1 matching was not required by the study design, and a larger control sample was allowed in order to improve the precision of case–control comparisons. Control participants were consecutively screened and recruited from four predefined sources: accompanying relatives of rheumatology outpatients, individuals presenting with non-inflammatory musculoskeletal complaints (mainly soft-tissue disorders), attendees of other hospital departments for non-musculoskeletal reasons, and individuals from the general population who were screened in the hospital setting but had no active healthcare engagement. These control sources were selected in order to assemble a comparison group from the same hospital setting while avoiding recruitment from a single background population. The same general inclusion process and exclusion criteria were applied to cases and controls in order to improve comparability and minimize selection bias.

To reduce confounding, cases and controls were excluded if they had medical conditions known to significantly influence fatigue or overall clinical status, including active malignancy, severe disability (wheelchair-bound), heart or respiratory failure, or chronic liver or kidney disease. Common comorbidities not directly associated with secondary fatigue—such as hypertension or dyslipidemia—were not considered exclusion criteria and were not systematically recorded. In addition, information on depression, anxiety, sleep disorders, fibromyalgia, and other chronic pain conditions was not systematically collected in either group. Although some of these conditions may have been documented in the clinical records of patients with axSpA, they were not available in a standardized manner and were therefore not included in the primary analyses.

All participants provided written informed consent prior to inclusion. The study protocol was approved by the local ethics committee (protocol code: PR329/22, approved 15 December 2022).

### 2.2. Study Variables

Sociodemographic data collected for all participants included sex and age. Sex was considered a key analytical variable and was used for stratified analyses. Anthropometric assessment included body mass index (BMI), which was calculated as weight in kilograms divided by height in meters squared and categorized according to standard World Health Organization criteria. Hemoglobin levels were recorded in both cases and controls, using the most recent available laboratory value, to allow for adjustment for anemia as a potential contributor to fatigue. For case–control comparisons, only variables available in both groups were considered.

Information on smoking status was collected and categorized as current smoker versus not current smoker. Engagement in regular physical activity was assessed by self-report and categorized dichotomously (yes/no) according to whether participants reported regular exercise. For descriptive purposes, patients who did not report regular exercise were classified as having a sedentary lifestyle. These lifestyle variables were only available in patients with axSpA and were not systematically recorded in the control population; accordingly, they were not included in the primary case–control comparisons and could not be considered potential confounders in between-group analyses. Socioeconomic variables such as education, income, and employment status were not systematically collected in either group.

In patients with axSpA, disease-related variables included disease duration, axSpA subtype (radiographic or non-radiographic), and HLA-B27 status. Musculoskeletal manifestations (peripheral arthritis, enthesitis, and dactylitis) and extra-articular manifestations (uveitis, psoriasis, onychopathy, and inflammatory bowel disease) were recorded. Current treatment was documented, including use of nonsteroidal anti-inflammatory drugs, glucocorticoids, conventional synthetic disease-modifying antirheumatic drugs, and biologic disease-modifying antirheumatic drugs. For biologic therapy, only current use at the time of data collection was recorded; specific agents and treatment duration were not systematically collected. These variables were retrieved from the medical records and corresponded to the most recent clinical evaluation available at the time of data collection.

Laboratory assessment in axSpA cases included hemoglobin and C-reactive protein (CRP); the most recent available values were used. Because the study was conducted in a real-world outpatient setting, laboratory parameters were obtained from routine clinical practice rather than from a protocol-mandated assessment. These measurements generally preceded the clinical visit at which FACIT-F and the other study variables were recorded by approximately 7 to 15 days, although the exact interval was not systematically collected.

Disease activity was evaluated using the Bath Ankylosing Spondylitis Disease Activity Index (BASDAI) [16] and the Axial Spondyloarthritis Disease Activity Score with CRP (ASDAS-CRP) [17], categorized according to established cut-offs for inactive, low, high, and very high disease activity. BASDAI is a patient-reported index ranging from 0 to 10, with higher scores indicating greater perceived disease activity. Patient-reported outcomes related to disease activity and impact included the Bath Ankylosing Spondylitis Functional Index (BASFI) [18] and patient global assessment (PGA; 0–10 numerical rating scale, 0 = best and 10 = worst). BASFI is a validated 10-item instrument assessing functional limitation in daily activities, with higher scores indicating worse physical function. Overall health status and disease impact were assessed using the Assessment of SpondyloArthritis International Society Health Index (ASAS-HI) [19], a validated instrument covering multiple domains of functioning and health. The ASAS-HI ranges from 0 to 17, with higher scores reflecting greater health impact and worse overall functioning.

Fatigue was assessed in all participants using the Functional Assessment of Chronic Illness Therapy–Fatigue (FACIT-F) questionnaire [20]. The FACIT-F generates a total score ranging from 0 to 52, with lower scores indicating greater fatigue severity and functional impact. Because no universally accepted FACIT-F threshold for clinically relevant fatigue has been established specifically for middle-aged and older adults with axSpA, we used a score below 40 to identify participants with at least mild fatigue according to previously proposed cross-sectional interpretation bands in axSpA [11]. In addition, fatigue severity was categorized according to proposed cross-sectional thresholds in axSpA, with scores > 40 indicating no fatigue or minimal fatigue, >30 to ≤40 indicating mild fatigue, >21 to ≤30 indicating moderate fatigue, and ≤21 indicating severe fatigue [11]. These categories were used to support descriptive interpretation of fatigue burdens and should not be interpreted as population-specific normative thresholds for middle-aged and older adults.

For case–control comparisons, analyses were restricted to variables available in both groups, including age, sex, BMI, hemoglobin levels, and FACIT-F scores. Disease-specific variables, inflammatory markers, lifestyle factors, disease activity indices, and functional measures were analyzed exclusively within the axSpA cohort.

### 2.3. Statistical Analysis

Sample size was determined by the availability of eligible participants during the study period. Continuous variables are presented as mean ± standard deviation and categorical variables as counts and percentages. Comparisons between axSpA cases and controls were performed using Student’s *t* test for continuous variables and the chi-square test or Fisher’s exact test for categorical variables, as appropriate. Primary analyses focused on fatigue severity assessed by FACIT-F scores. FACIT-F scores were additionally summarized across ASDAS-CRP disease activity categories (inactive, low, high, and very high) and compared descriptively across categories. Correlations between FACIT-F scores and key clinical variables were assessed using Spearman correlation coefficients.

We assessed the completeness of the main study variables descriptively. Descriptive summaries were based on available data for each variable, and between-group comparisons and regression models were performed using complete cases for the variables included in each analysis; no imputation was performed.

All case–control analyses were conducted for the overall population and stratified by sex. The magnitude of between-group differences in fatigue was quantified using standardized mean differences (Cohen’s d).

Within the axSpA cohort, associations between fatigue and clinical, laboratory, and disease-related variables were examined using correlation analyses and sex-stratified multivariable linear regression models, with age included as a covariate. Correlations among the main patient-reported predictors considered for the multivariable models were examined, and variance inflation factors (VIFs) were calculated in the reduced sex-stratified models to assess potential collinearity. To limit overfitting in the sex-stratified analyses, reduced multivariable linear regression models including age, BASDAI, and ASAS-HI were fitted. These variables were selected to represent age, perceived disease activity, and overall health impact. All multivariable analyses were conducted using complete cases.

All statistical analyses were performed using R software, version 4.5.1. Two-sided *p* values < 0.05 were considered statistically significant.

### 2.4. Ethical Considerations

The study was conducted in accordance with the Declaration of Helsinki and was approved by the Ethics Committee of Hospital Universitari de Bellvitge (reference: PR329/22). All participants provided written informed consent prior to inclusion.

## 3. Results

A total of 173 patients with axSpA (120 men and 53 women) and 383 control subjects were included in the analysis. The mean age of patients was 64.2 ± 9.5 years, with no significant differences between men and women. Women reported a later age at symptom onset and had lower body weights and heights than men, while body mass index did not differ between sexes. Disease-related characteristics, laboratory parameters, and treatments are detailed in Table 1. Missingness was low (<5%) for most key patient variables.

Fatigue was highly prevalent among patients with axSpA. Using a FACIT-F cut-off value of <40 as an operational threshold for at least mild fatigue, women more frequently met this criterion than men (84.9% vs. 50.0%, *p* < 0.001). Mean FACIT-F scores were markedly lower in women, indicating more severe fatigue (29.4 ± 10.4 vs. 37.4 ± 10.2; *p* < 0.001). These differences were accompanied by a consistently higher disease burden in women, including higher perceived disease activity (BASDAI), worse functional status (BASFI), higher patient global assessment (PGA) scores, and greater health impact (ASAS-HI) (Table 1).

In case–control comparisons, patients with axSpA showed significantly greater fatigue burdens than controls, both overall and after sex stratification (Table 2). Mean FACIT-F scores were lower in patients than in controls, and the proportion with FACIT-F scores < 40 was also consistently higher. This pattern was observed in both men and women, with descriptively larger differences among women.

**Figure 1:**
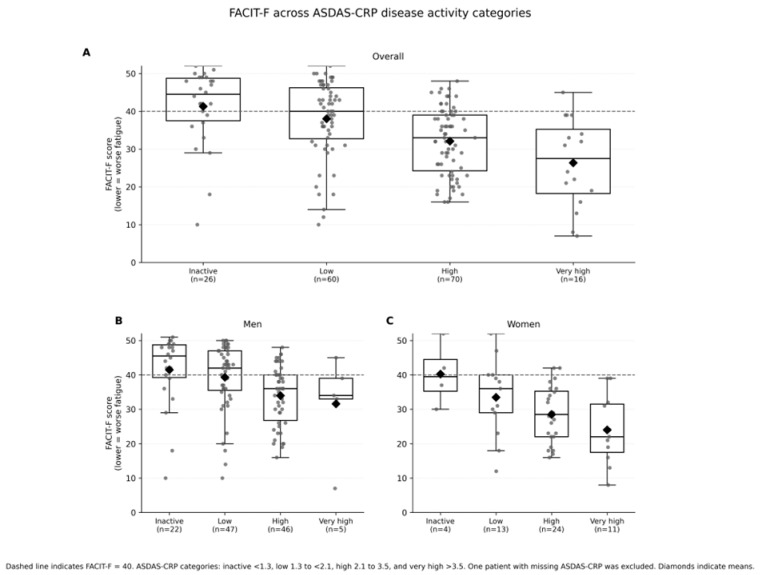
FACIT-F scores according to ASDAS-CRP disease activity categories in the overall cohort and by sex. The upper panel (**A**) shows FACIT-F scores across ASDAS-CRP disease activity categories in the overall axSpA cohort, whereas the lower panels (**B**,**C**) show the corresponding sex-stratified analyses in men and women. Lower FACIT-F scores indicate greater fatigue. ASDAS-CRP disease activity categories were defined as inactive (<1.3), low disease activity (1.3 to <2.1), high disease activity (2.1–3.5), and very high disease activity (>3.5). Boxplots show the median and interquartile range; dots represent individual participants and diamonds indicate mean values. The dashed horizontal line indicates the threshold of FACIT-F score < 40, consistent with clinically relevant fatigue.

Severe fatigue (FACIT-F score ≤ 21) was observed in 24.5% of women compared with 10.8% of men (*p* < 0.02). FACIT-F scores progressively worsened across ASDAS-CRP disease activity categories. Mean FACIT-F values were 41.3 in inactive disease, 38.1 in low disease activity, 32.1 in high disease activity, and 26.4 in very high disease activity. This gradient was also observed in sex-stratified analyses, with decreasing FACIT-F scores across increasing ASDAS-CRP categories in both men and women (Figure 1).

Correlations between FACIT-F score and key clinical variables are presented in Table 3. The strongest association was observed for ASAS-HI, followed by BASFI, BASDAI, PGA, and ASDAS-CRP, whereas correlations with age, BMI, and CRP were weak or non-significant overall.

In sex-stratified analyses, fatigue scores were significantly lower in patients than in controls in both men and women (*p* < 0.001 for both comparisons). The absolute difference in fatigue severity was greater among women: female patients exhibited mean FACIT-F scores 10.9 points lower than female controls, whereas the corresponding difference among men was 5.9 points (Table 4). Consistently, standardized effect sizes indicated a moderate difference in men and a large difference in women (Table 4), supporting that fatigue associated with axSpA were higher than those observed in the control population, with larger descriptive differences among women.

In multivariable analysis, the models included 110 men and 51 women with complete data. ASAS-HI was independently associated with FACIT-F score in both men (β: −1.74, 95% CI: −2.08 to −1.41; *p* < 0.001) and women (β: −1.80, 95% CI: −2.35 to −1.26; *p* < 0.001), whereas BASDAI only showed an additional association in women (β: −1.19, 95% CI: −2.09 to −0.30; *p* = 0.010) but not in men (β: −0.44, 95% CI: −1.13 to 0.26; *p* = 0.213).

## 4. Discussion

The present case–control study provides a comprehensive characterization of fatigue in a cohort of middle-aged and older adults with axSpA. We found that fatigue was highly prevalent and clinically relevant, affecting a substantial proportion of patients, and that its burden was higher than in an age- and sex-frequency-matched control population. These findings indicate that fatigue in axSpA is unlikely to be fully accounted for by background fatigue levels and constitutes a major component of disease impact in this age group.

A key and novel observation was the marked sex-specific pattern of fatigue burden. Women with axSpA experienced more severe fatigue than men, both in absolute terms and relative to their respective control populations. The larger gap observed between female patients and female controls suggests that excess fatigue associated with axSpA may be particularly pronounced among women. Importantly, these differences were observed in patients with established disease and long-standing exposure to contemporary therapies, emphasizing the persistence and clinical relevance of fatigue despite advances in inflammatory disease control [8].

Previous studies have consistently reported a high prevalence of fatigue in axSpA, supporting its relevance as a major contributor to disease burden [7]. However, most available data derive from cross-sectional analyses conducted within patient cohorts, with considerable heterogeneity in study populations, assessment instruments, and analytical approaches. In the absence of appropriate external comparators, the clinical interpretation of fatigue severity has therefore remained limited [6,7]. Recent systematic reviews have highlighted this gap, identifying a paucity of case–control data examining fatigue in axSpA [7]. By directly comparing patients with an age- and sex-matched control population, the present study addresses this limitation and shows that fatigue levels in axSpA were higher than those observed in the control population, particularly in older individuals. Nevertheless, because socioeconomic characteristics and physical activity were not available in a standardized manner for both groups, some residual confounding in the interpretation of case–control differences cannot be excluded. The magnitude of these differences also appears clinically meaningful. According to the interpretation framework proposed by Cella et al. [11] for FACIT-F scores in axSpA, between-group differences of approximately 2.14–5.34 points may be considered meaningful, whereas within-patient improvement has been estimated in the range of 5–11 points. In this context, the observed patient–control differences of 5.9 points in men and 10.9 points in women support the clinical relevance of the excess fatigue burden identified in our study.

The pronounced sex differences observed are consistent with findings in other inflammatory rheumatic diseases, in which women frequently report higher symptom burdens and worse patient-reported outcomes [8,21]. Potential contributing factors include differences in pain perception, functional impairment, and psychosocial factors, as well as a greater subjective impact of symptoms despite comparable levels of objective inflammation [8,21,22]. The persistence of sex differences relative to controls suggests that these findings cannot be attributed solely to background differences between women and men in the control population.

Within the axSpA cohort, fatigue severity progressively worsened across ASDAS-CRP disease activity categories, whereas in the reduced multivariable models, measures of overall health impact showed a stronger and more consistent association with fatigue than did inflammatory markers. This pattern supports the concept that fatigue reflects a multidimensional aspect of disease burden rather than serving as a direct surrogate of inflammatory activity [23,24]. At the same time, because the exact interval between blood sampling and fatigue assessment was not systematically recorded, some temporal mismatch cannot be excluded and may have attenuated the observed association with CRP. In particular, the robust association with the ASAS Health Index supports the interpretation of fatigue as a marker of overall disease impact captured by a multidomain patient-reported outcome measure [24].

From a clinical perspective, these findings highlight that control of inflammatory activity alone may be insufficient to address the full burden experienced by patients with axSpA. The high prevalence and severity of fatigue observed, even among patients receiving contemporary therapies, underscore the need to consider fatigue as a distinct and clinically relevant outcome. The use of validated instruments such as the FACIT-F may facilitate a more comprehensive assessment of patient well-being and help identify individuals who could benefit from targeted, multidisciplinary interventions beyond anti-inflammatory treatment [9]. The FACIT-F severity bands used in this study were based on recently proposed cross-sectional thresholds in axSpA and were intended to support descriptive interpretation of fatigue burden; however, their applicability as population-specific normative thresholds for middle-aged and older adults warrants cautious interpretation.

Several limitations should be acknowledged. The cross-sectional design precludes causal inference, and the study was conducted at a single tertiary center. In addition, certain psychosocial and non-inflammatory factors that may influence fatigue were not systematically collected. The hospital-based selection of controls from different recruitment sources may have introduced heterogeneity in background fatigue levels, and the potential influence of this variability could not be formally assessed in subgroup analyses. Although similar inclusion and exclusion criteria were applied across groups, this aspect should be considered when interpreting the observed case–control differences, as it may have led to a conservative estimate of the true fatigue gap between patients and controls. In addition, patients had a higher mean BMI than controls, and this difference may have contributed to the observed case–control differences in fatigue. Physical activity was also only recorded in patients with axSpA and was not available in controls, precluding adjustment for this potentially relevant determinant of fatigue in the case–control analyses. Although sedentary lifestyle was reported in 23.7% of patients, its potential contribution to the observed fatigue differences cannot be excluded. An additional limitation is the relatively small number of women with axSpA, which may have reduced the precision of sex-stratified multivariable analyses, increased the risk of overfitting, and limited power for subgroup-specific inference. Accordingly, the findings from female-specific multivariable models should be interpreted as exploratory. Finally, the higher hemoglobin levels observed in male patients compared with male controls should also be interpreted cautiously, as laboratory values were derived from routine clinical practice and may have been influenced by unmeasured factors such as smoking, hydration status, timing of blood sampling, or characteristics of the control population.

The present study also has several strengths. First, the case–control design with an external comparator allowed us to place the observed fatigue burden in axSpA in a broader clinical context and to estimate the extent of the difference in fatigue levels compared to similarly aged individuals without inflammatory arthritis. Second, the study specifically focused on middle-aged and older adults, a clinically relevant population in whom fatigue may be shaped by the combined effects of longstanding disease, functional limitation, and age-related factors, yet who remain underrepresented in the literature. Third, fatigue was assessed using the FACIT-F, a validated instrument with good psychometric performance in axSpA and clinically interpretable thresholds that facilitated both quantitative and descriptive interpretation of fatigue severity. Finally, the sex-stratified analytical approach enabled the identification of a clear sex-related gradient in fatigue burden while also highlighting the need for cautious interpretation and further confirmation in larger female samples.

Future studies should further examine the relative contribution of inflammatory, functional, psychosocial, and comorbidity-related factors to fatigue in axSpA, particularly in middle-aged and older adults. Interventional research will also be needed to determine whether systematic fatigue assessment and targeted management strategies translate into improved patient outcomes.

## 5. Conclusions

Fatigue in axSpA constitutes a substantial and clinically relevant component of disease burden in middle-aged and older adults. Compared with an age- and sex frequency-matched control population, fatigue levels were significantly higher, with descriptively larger differences observed in women. These findings indicate that fatigue represents a distinct dimension of disease impact that is not fully captured by conventional measures of inflammatory activity and support the rationale for its systematic evaluation in routine clinical care.

## Figures and Tables

**Table 1:** Description of the variables in the analyzed population.

Variable	Total n = 173	Men n = 120	Women n = 53	*p* Value
Age	64.24 (9.48)	64.87 (9.75)	62.83 (8.77)	0.177
Age at symptom onset	32.89 (15.01)	30.76 (14.14)	37.75 (15.92)	0.008
Weight	77.72 (14.99)	80.16 (13.82)	72.23 (16.15)	0.003
Height	163.68 (9.77)	165.68 (9.04)	159.19 (9.94)	<0.001
BMI (kg/m^2^)	29 (5.07)	29.27 (5.03)	28.39 (5.15)	0.303
Sedentary lifestyle	41 (23.7)	28 (23.3%)	13 (24.5%)	0.878
Current smoker	49 (28.32)	34 (28.8%)	15 (28.3%)	0.945
BASDAI	3.81 (2.21)	3.28 (1.98)	5.03 (2.24)	<0.001
BASFI	4.20 (2.69)	3.92 (2.75)	4.85 (2.45)	0.028
PGA	3.88 (2.3)	3.53 (2.25)	4.68 (2.23)	0.002
ASDAS-CRP	2.20 (1)	1.99 (0.82)	2.67 (1.2)	<0.001
ASAS-HI	6.68 (4.43)	5.92 (4.53)	8.33 (3.76)	<0.001
HLA-B27 (+)	132 (76.3%)	97 (80.8%)	35 (66%)	0.108
CRP (mg/L)	4.09 (8.44)	4.32 (9.79)	3.57 (4)	0.475
Radiographic axSpA	129 (75%)	96 (80%)	33 (63.5%)	0.010
Family history of spondyloarthritis	35 (20.3%)	22 (18.5%)	13 (24.5%)	0.482
Peripheral arthritis	70 (40.5%)	52 (43.3%)	18 (34%)	0.322
Psoriasis	22 (12.7%)	15 (12.5%)	7 (13.2%)	1.000
Inflammatory bowel disease	17 (9.8%)	11 (9.2%)	6 (11.3%)	0.872
Use of biologic agents	92 (53.2%)	62 (51.7%)	30 (56.6%)	0.62

Values are expressed as mean (SD) or n (%). ASAS-HI: Assessment of SpondyloArthritis International Society Health Index; ASDAS-CRP: Axial Spondyloarthritis Disease Activity Score with CRP; BASDAI: Bath Ankylosing Spondylitis Disease Activity Index; BASFI: Bath Ankylosing Spondylitis Functional Index; BMI: body mass index; CRP: C-reactive protein; PGA: patient global assessment.

**Table 2:** Comparison between patients and controls.

	Variable	Patients	Controls	*p* Value
Total		n = 173	n = 383	
	FACIT-F	34.9 (10.9)	41.5 (9.1)	<0.001
	FACIT-F < 40	105 (60.7%)	119 (31.1%)	<0.001
	BMI (kg/m^2^)	29.0 (5.1)	27.3 (4.8)	<0.001
	Hemoglobin	146 (15.5)	140.3 (13.9)	<0.001
Men		n = 120	n = 157	
	FACIT-F	37.4 (10.2)	43.3 (8.3)	<0.001
	FACIT-F < 40	60 (50%)	38 (24.2%)	<0.001
	BMI (kg/m^2^)	29.3 (5)	27.2 (4.2)	<0.001
	Hemoglobin	150.3 (15.5)	146.9 (15.1)	0.088
Women		n = 53	n = 226	
	FACIT-F	29.4 (10.4)	40.3 (9.5)	<0.001
	FACIT-F < 40	45 (84.9%)	81 (35.8%)	<0.001
	BMI (kg/m^2^)	28.4 (5.1)	27.5 (5.2)	0.242
	Hemoglobin	136.3 (10.3)	136.3 (11.4)	0.968

Values are expressed as mean (SD). BMI: body mass index; FACIT-F: Functional Assessment of Chronic Illness Therapy–Fatigue questionnaire.

**Table 3:** Correlations between FACIT-F score and key clinical variables.

Variable	Overall	Men	Women
Age	−0.00 (0.968)	−0.11 (0.232)	0.07 (0.629)
BMI	−0.02 (0.800)	−0.07 (0.442)	0.03 (0.858)
Hemoglobin	0.27 (<0.001)	0.21 (0.020)	−0.21 (0.139)
CRP	−0.08 (0.305)	−0.00 (0.971)	−0.27 (0.054)
BASDAI	−0.57 (<0.001)	−0.51 (<0.001)	−0.48 (<0.001)
BASFI	−0.59 (<0.001)	−0.56 (<0.001)	−0.65 (<0.001)
PGA	−0.43 (<0.001)	−0.38 (<0.001)	−0.30 (0.031)
ASDAS-CRP	−0.47 (<0.001)	0.40 (<0.001)	−0.42 (0.002)
ASAS-HI	−0.79 (<0.001)	−0.77 (<0.001)	−0.75 (<0.001)

BMI: body mass index; CRP: C-reactive protein; BASDAI: Bath Ankylosing Spondylitis Disease Activity Index; BASFI: Bath Ankylosing Spondylitis Functional Index; PGA: patient global assessment; ASDAS-CRP: Axial Spondyloarthritis Disease Activity Score with CRP; ASAS-HI: Assessment of SpondyloArthritis International Society Health Index.

**Table 4:** Differences in FACIT-Fatigue scores and effect size by sex.

FACIT-F	Men				Women			
	Patients (n: 120)	Controls (n: 157)	Difference (axSpA-Controls)	Cohen’s d	Patients (n: 53)	Controls (n: 226)	Difference (axSpA-Controls)	Cohen’s d
	37.4 ± 10.2	43.3 ± 8.3	−5.9	−0.64	29.4 ± 10.4	40.3 ± 9.5	−10.9	−1.13

## Data Availability

The datasets used and analyzed during the current study are available from the corresponding author upon reasonable request.
